# Investigating the clinical utility of plasma succinate with insights from an *Sdhb*-deficient murine model

**DOI:** 10.1530/ERC-26-0323

**Published:** 2026-10-13

**Authors:** Yasemin C Cole, Ifat Abramovich, Jonatan Fernandez-Garcia, France Docquier, James MacFarlane, Xia Xu, Thorkell Andresson, Iris Indig, Herui Wang, Anagha Krishnan, Shuran Chen, Ben Challis, Eyal Gottlieb, Zhengping Zhuang, Eamonn R Maher, Ruth T Casey

**Affiliations:** ^1^ Department of Genomic Medicine, University of Cambridge, Cambridge Biomedical Campus, Cambridge, UK; ^2^ Neuro-Oncology Branch, National Cancer Institute, National Institutes of Health, Bethesda, Maryland, USA; ^3^ Technion Integrated Cancer Center, Israel Institute of Technology, Haifa, Israel; ^4^ Department of Endocrinology, Cambridge University Hospital, Cambridge Biomedical Research Centre, Addenbrooke’s Hospital, Cambridge, UK; ^5^ Protein Characterization Laboratory, Cancer Research Technology Program, Frederick National Laboratory for Cancer Research, Leidos Biomedical Research, Inc., Frederick, Maryland, USA; ^6^ Laboratory for Integrative Cancer Immunology, National Cancer Institute, National Institutes of Health, Bethesda, Maryland, USA; ^7^ Aston Medical School, College of Health and Life Sciences, Aston University, Birmingham, UK

**Keywords:** *SDHB,* paraganglioma/pheochromocytoma, metabolomics, liquid biopsy

## Abstract

The succinate dehydrogenase (SDH) enzyme, composed of four subunits (A–D), has a key role in the Krebs cycle and oxidative phosphorylation. Germline pathogenic variants (GPVs) in the genes encoding the four subunits of the succinate dehydrogenase (SDH) enzyme (*SDHA*/*SDHB*/*SDHC*/*SDHD*), collectively known as *SDHx*, are recognized as a paradigm for the role of disordered metabolism in oncogenesis as GPVs in *SDHx* lead to a truncated citric acid cycle due to reduced or absent function of the SDH enzyme and accumulation of the oncometabolite succinate. GPVs in *SDHx* are the most common cause of hereditary PPGL and are associated with a higher risk of malignant PPGL and predispose to other tumors including renal cell carcinoma, gastrointestinal stromal tumors (GIST), and pituitary adenomas. Utilizing the linkage of *SDHx* to metabolic dysfunction, we performed prospective plasma metabolomics and identified succinate as a biomarker for early diagnosis of an underlying *SDHx* variant. Succinate reflected *SDHx* deficiency in individuals with germline predisposition and a small number of patients with somatic *SDHx* deficiency, and succinate levels correlated with tumor burden. Longitudinal sampling of patients illustrated that serial succinate measurements might be used as a biomarker for disease surveillance. These findings were validated by tissue analysis in a mouse model of *Sdhb* deficiency, where elevated succinate was observed in adrenal glands. While circulating plasma levels did not mirror the human cohort, this discrepancy suggests specific cellular thresholds for succinate or *SDHx* deficiency in adrenal gland tissue and highlights species-specific metabolic regulation.

## Main text

A renewed interest in cancer metabolism, considered to be one of the hallmarks of cancer, was fostered by the discovery of succinate dehydrogenase (*SDHx*) gene alterations in pheochromocytoma (PCC) and paraganglioma (PGL) in the early 2000s. Currently, more than 20 genes have been associated with PPGL development (1), and nearly half of the PPGL susceptibility genes are implicated in metabolic pathways. At an annual incidence of 2–6 per million people per year (2), these neuroendocrine tumors are frequently inherited due to a germline predisposition, the most common of which is due to GPVs in *SDHB*. All PPGLs are now considered to have malignant potential under the recent WHO guidelines, and tumors bearing *SDHx* GPVs confer one of the highest metastatic risk potentials and a lifelong risk of multifocal PPGL. Early diagnosis of a GPV in *SDHx* can facilitate stratified surveillance protocols, early diagnosis, appropriate surgical management, and timely family screening.

As a mediator between the TCA cycle and electron transport chain (ETC) in the inner mitochondria, the SDH complex plays an integral role in metabolic homeostasis, and the disruption of this complex results in a truncated TCA cycle. *SDHx* PV-harboring cancers are the quintessential example of Warburg’s theory of cancer cell metabolism driven by endogenous genetic factors. Even when oxygen levels are sufficient, *SDHx*-deficient cancer cells upregulate hypoxia pathways, termed pseudohypoxia, leading to both metabolic and epigenetic rewiring (3, 4).

A variety of diagnostic tests for the early detection of *SDHx* deficiency have been described, including SDHB immunohistochemistry, to identify *SDHx*-deficient tumors, *in vitro* measurements of succinate or succinate-to-fumarate ratio (SFR) in tumor tissue (5), and development of MRI spectroscopy techniques to facilitate *in vivo* detection of oncometabolites, including succinate and fumarate in patients with *SDHx* and *FH* PVs have also been reported (6, 7, 8).

These described techniques are based on the premise that tumor tissue will have biallelic loss of function of the respective citric acid cycle gene, leading to disruption of the encoded enzyme in keeping with Knudson’s ‘two-hit’ hypothesis. A loss of SDHB staining on immunohistochemistry is a validated method for diagnosing PVs in the *SDHx* genes and can provide a rapid assessment of pathogenicity for a novel *SDHx* variant of uncertain significance (9). Furthermore, this technique may subsequently lead to the diagnosis of an underlying GPV, leading to cascade genetic testing of family members. Notably, SDHB protein expression is typically demonstrated in adjacent normal tissue even if absent in tumoral cells (6), in keeping with the hypothesis that biallelic inactivation is required to disrupt the SDH enzyme complex. Intriguingly, two recent studies have reported that serum succinate levels are elevated in patients with *SDHx* GPVs and PPGL, as well as in asymptomatic carriers (10, 11). This finding suggests that i) succinate accumulation is not dependent on cellular biallelic inactivation or ii) that clusters of cellular biallelic inactivation accumulate in tissues over a patient’s lifetime. Both theories require further interrogation as a better understanding of the pathological mechanism underpinning this observation could improve our prediction of disease penetrance in patients carrying PVs in *SDHx* and may reveal therapeutic or preventative medicine strategies.

Our studies aim to extend the findings of earlier studies by examining succinate levels in a well-characterized cohort of 122 individuals (both asymptomatic *SDHx* PV carriers and those with tumors) and examine the mechanistic link between tissue-level loss of SDH function and systemic metabolomic shifts using an *Sdhb*-deficient murine model.

## Methods

### Participant recruitment and clinical data

Patients with a current or past diagnosis of PPGL or germline susceptibility to a PPGL were recruited and consented to the Molecular Pathology of Human Genetic Disease Study (South Birmingham REC CA/125) at Addenbrooke's Hospital. Of 312 eligible participants, 122 provided written, informed consent at Addenbrooke’s neuroendocrine tumor clinic during the prospective study (2020–2024), in accordance with the principles of the Declaration of Helsinki.

Participants were managed according to standard clinical management guidelines, such as imaging at 6 months post-surgery and plasma metanephrine measurements post-surgical resection or annual measurement of plasma metanephrines in asymptomatic carriers of a PPGL predisposition gene. Individuals with a GPV in an *SDH* subunit gene were deemed ‘*SDHx*’ and those with a non-*SDHx* PPGL predisposition gene or negative germline genetics were indicated as ‘*non-SDHx’*. When possible, serial plasma samples were obtained from participants during follow-up appointments. Clinical and analytic variables were collected, including quantitative plasma-free metanephrine levels on the same day or closest to the time of blood collection, patient age and sex, tumor size (maximum diameter), past tumor, tumor location (adrenal/extra-adrenal PGL), germline/somatic genetic testing results, SDHB immunohistochemistry, BMI, weight, and medications at the time of plasma collection were extracted from the clinical records. Plasma metanephrine (normetanephrine, metanephrine, and 3-methoxytyramine) levels, histopathological reports, and CT/MRI and/or functional imaging were reviewed to categorize patients into groups based on their genetic and tumor status. The definition of benign and metastatic disease is defined in the Supplementary Methods (see section on Supplementary materials given at the end of the article). False elevation of metanephrines due to interfering medications, inappropriate fasting or posture at the time of blood draw, and catecholamine-containing foods was noted. Two samples were excluded at the time of analysis due to the subsequent identification of a non-PPGL syndrome-associated cancer upon further screening, leaving 185 samples for analysis.

### Clinical sample collection and processing

Blood samples (*n* = 187) from patients were collected in lithium heparin tubes and placed on ice. Samples were centrifuged for 15 min at 2,000 *g* at 4°C. The supernatant was transferred into cryovials, immediately snap-frozen on dry ice, and stored at −80°C until extraction. Plasma samples were obtained at the same time as clinical plasma metanephrines.

### Murine plasma and tissue collection with central carbon metabolite assay

Experimentation was conducted using two Th-Cre-driven *Sdhb*-deficient murine models, with details of husbandry, genotyping, and tissue collection provided in the Supplementary Methods.

### Human plasma liquid chromatography–mass spectrometry and targeted metabolomics

Extracted plasma metabolites from human samples were analyzed using an ultra-high-performance liquid chromatography coupled to high-resolution mass spectrometry. Metabolites were identified by the exact mass of the singly charged ion and by known retention time(s) using an in-house MS library built by running commercial standards of all detected metabolites. The LC-MS/MS method employed was adapted from previous publications (12, 13). For additional information, please see Supplementary Methods.

### Data analysis and statistics

For participants with multiple plasma samples, the first sample was utilized, unless otherwise noted. To assess group differences, metabolite peak intensities and concentrations were analyzed using Excel, MetaboAnalyst (version 6.0, https://www.metaboanalyst.ca/), and Prism version 10.1.1. For targeted metabolomic analysis, AUROC curves were performed using the biomarker analysis module on MetaboAnalyst, and the optimal cutoff was determined by MetaboAnalyst’s ROC module. Statistical tests were performed in Prism version 10.1.1. Pearson r correlation and/or Spearman *ρ* was generated to assess the correlation of succinate with age, BMI, and tumor size, and data were plotted using a simple linear regression. Given the right-skewed distribution of benign tumor sizes, plasma succinate and tumor size were natural log-transformed prior to correlation analysis. The succinate–tumor size relationship was assessed by Pearson correlation and ordinary least-squares linear regression. All tests were two-tailed, and a *P*-value <0.05 was deemed statistically significant.

## Results

### Patient cohort characteristics

One hundred twenty-two participants consented to the study, from whom 185 samples were collected during a two-year period (November 2020–May 2022). Patients were categorized by *SDHx* gene status (germline and somatic *SDHx*, *n* = 61; *non-SDHx*, *n* = 61), tumor status (PGL, *n* = 9; HNPGL, *n* = 15; GIST, *n* = 12; renal oncocytoma, *n* = 1; neuroblastoma, *n* = 1; and PCC, *n* = 13), and metastatic disease (*n* = 23) (Fig. 1, Table 1). The average age of participants was 45.68 (range: 17–81), with a younger age observed in *SDHx* carriers (38.49 years) and a male-to-female ratio of 1.5:1 in the overall cohort versus 1.3:1 in *SDHx* carriers (Table 1). Patients were prospectively followed for recurrence and metastasis along with response to surgical and radiotherapy from November 2020 to July 2024.

**Figure 1:**
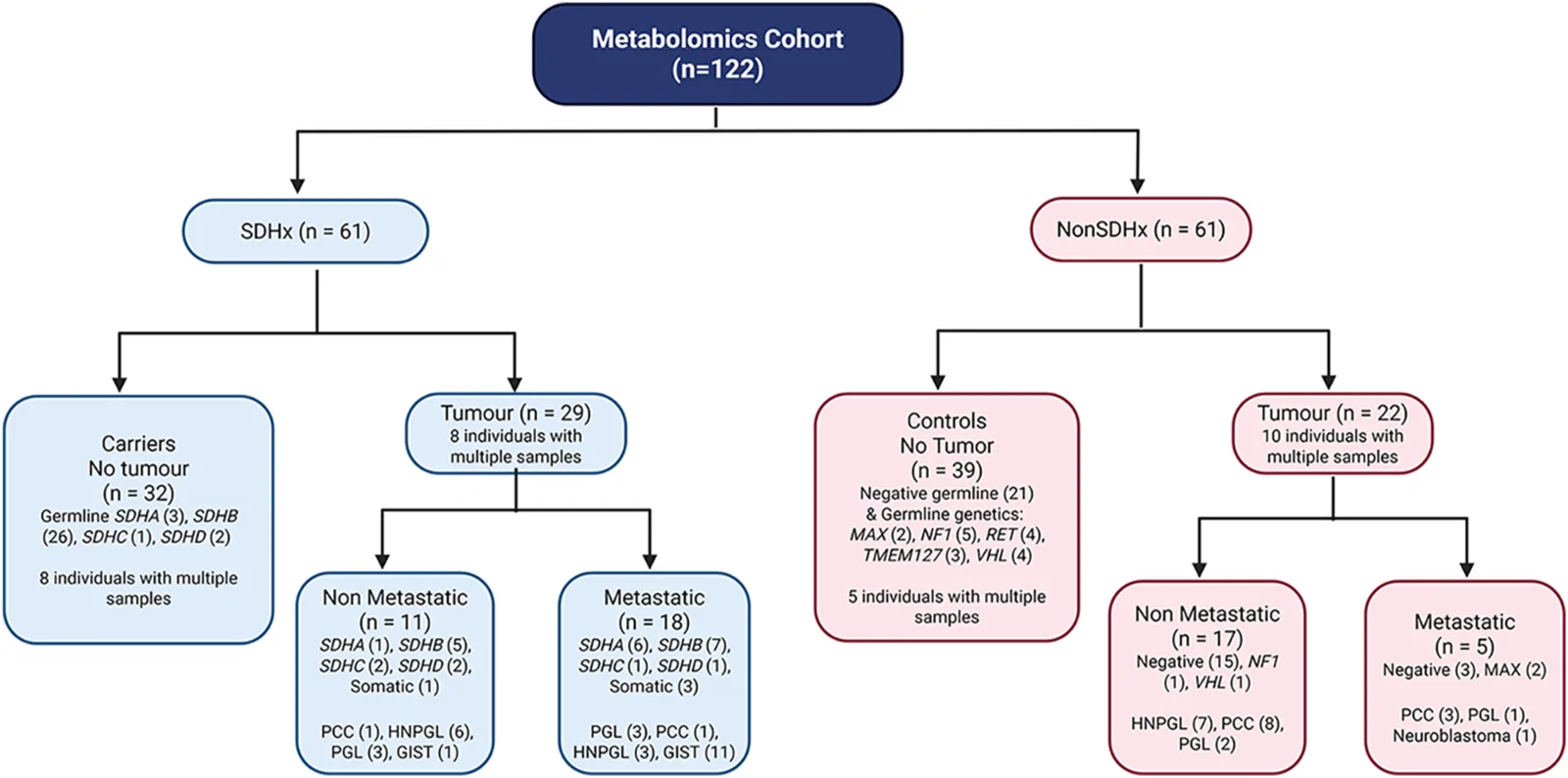
Study design of patient cohort. The diagram illustrates a breakdown of 122 participants with *SDHx* deficiency (blue) or *non-SDHx* genetics (red) (negative germline genetics or another PPGL-associated gene, except for *SDHx*). Subgroups are broken down into individuals with or without tumors, and into metastatic and non-metastatic tumors, with the number of individuals with each gene and the number of individuals with each tumor subtype in parentheses. Individuals with multiple samples are denoted in the appropriate boxes.

**Table 1:** Metabolomics cohort demographics and tumor phenotype.

Category	Participants (*n*) (% of cohort)	Tumors	Mean age (range)	Female:male (ratio)
Total cohort	122	NA	45.68 (17–81)	73:49 (1.5:1)
*SDHx* carriers	32 (26.2%)	NA	38.49 (17–76)	18:14 (1.3:1)
*Non-SDHx* controls (no tumor at testing)	39 (32.0%)	NA	52.27 (19–81)	25:14 (1.8:1)
Non-metastatic *SDHx*-deficient tumors	11 (9%)	PCC (1), HNPGL (6), PGL (3), GIST (1)	44.17 (21–73)	7:4 (1.75:1)
Non-metastatic *non-SDHx* tumors	17 (13.9%)	HNPGL (7), PCC (8), PGL (2)	50.36 (28–77)	10:7 (1.42:1)
Metastatic *SDHx* tumors	18 (14.8%)	PGL (3), PCC (1), HNPGL (3), GIST (11)	41.83 (18–74)	9:9 (1:1)
Metastatic *non-SDHx* tumors	5 (4.1%)	PCC (3), PGL (1), neuroblastoma (1)	41.47 (18–69)	4:1 (4:1)

Summary demographics of each subgroup (*SDHx* vs *non-SDHx*; no tumor vs tumor; metastatic vs non-metastatic) are provided for the metabolomics cohort. *Non-SDHx* category consists of individuals with either negative germline genetics or identification of a pathogenic variant in a PPGL-associated gene apart from *SDHx*. Tumor subtype was determined by imaging and/or histology; age is defined by the age when the patient was first recruited.

Of the plasma samples, 48.1% (89/185) were from 71 asymptomatic participants who had no evidence of PPGL at the time of plasma collection (based on concurrent interval imaging and plasma metanephrine testing). Of these 71 asymptomatic participants, 32 were asymptomatic carriers (19 *SDHx*; 13 *non-SDHx*) with no current or previous history of PPGL, and 39 had a history of PPGL (13 *SDHx*; 26 *non-SDHx*). Figure 1 graphically depicts the cohort of individuals based on *SDHx* status, tumor type and presence, and metastatic disease (*n* = 23). Longitudinal samples were collected from a subset of patients (13 asymptomatic gene carriers and 18 patients with tumors, with or without treatment, to assess disease progression) (Fig. 1).

### Plasma succinate distinguishes *SDHx *carriers from non-carriers and correlates with age and BMI

Targeted metabolomics profiling (129 metabolites) was performed on plasma samples by LC–MS. Analysis focused on succinate concentrations given the significance of this metabolite identified in prior publications and direct connection with *SDHx* deficiency. Comparing succinate levels in asymptomatic *SDHx* carriers (*n* = 32) and *non-SDHx* carriers (*n* = 39), *SDHx* carriers had significantly higher plasma succinate levels compared with controls (adjusted *P*-value = 0.0392). AUROC curve analysis of succinate concentrations between asymptomatic carriers demonstrated that a cutoff of 3.69 μM provided an ROC of 0.825 (sensitivity 75%, specificity 79.5%) (Fig. 2, Panels A and B). Individuals harboring *SDHA* and *SDHB* (*n* = 36) GPVs had higher succinate compared with *SDHC* and *SDHD* GPV carriers (*n* = 4) (*P*-value = 0.0255, two-tailed Mann–Whitney test) (Fig. 2, Panel C). Succinate was not significantly different in carriers harboring *SDHB* missense versus nonsense GPVs (two-tailed Mann–Whitney test, *P*-value = 0.9064). Comparing carriers with *SDHB* amorphic vs hypomorphic variants, classified by their predisposition to HNPGLs or PPGLs, as outlined by Lee *et al.* (14), there was no significant difference in succinate levels (Supplementary Fig. 1). Evaluation of clinical variables impacting succinate concentrations, such as age, gender, and BMI, revealed that age was associated with elevated succinate in *non-SDHx* females (see Supplementary Fig. 2D and E).

**Figure 2:**
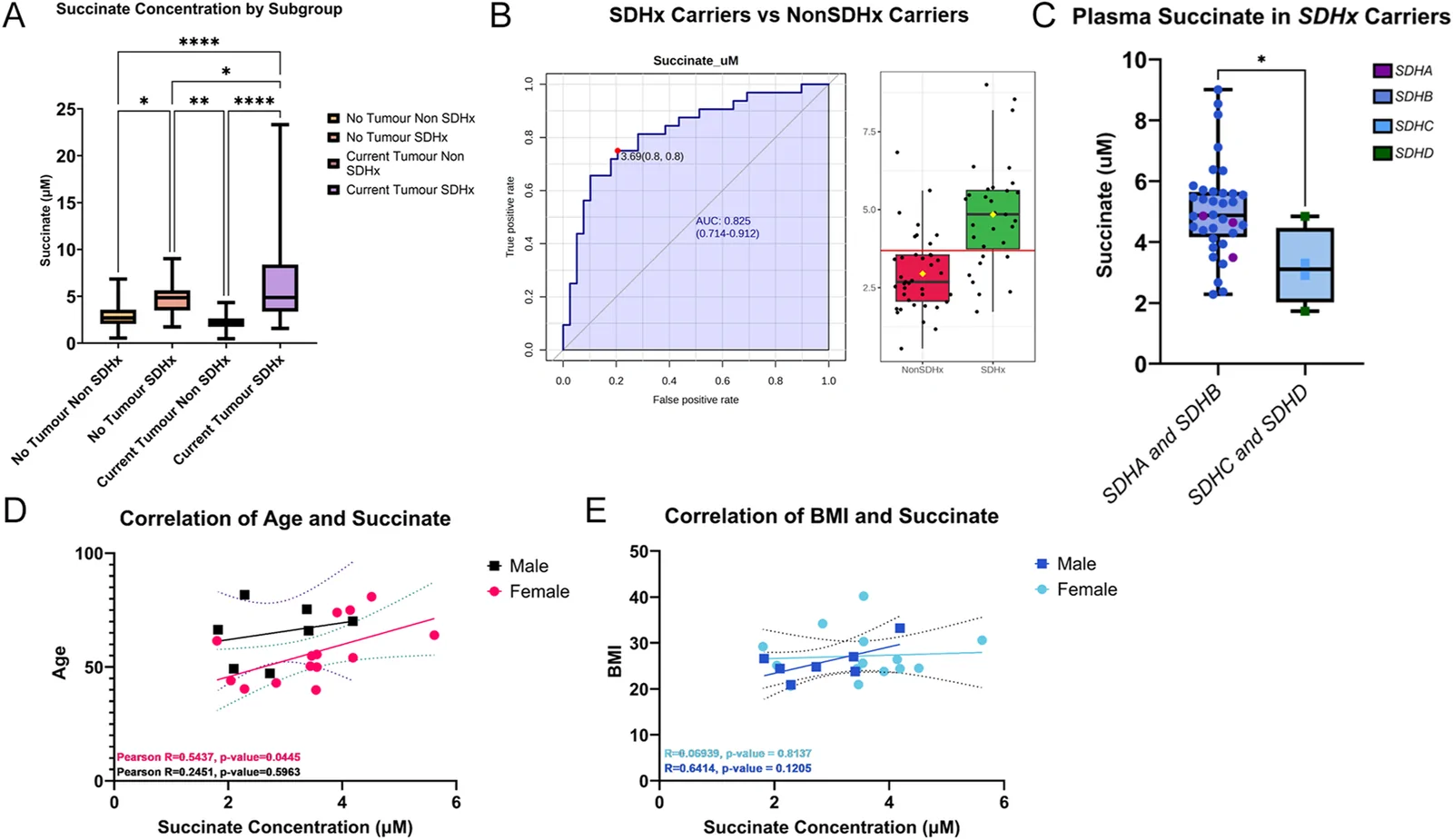
Elevated succinate in asymptomatic *SDHx* carriers and correlation with sex, age, and BMI. (Panel A) Succinate in *SDHx* carriers with and without tumors is significantly elevated compared with *non-SDHx* carriers without tumors (one-way ANOVA, Tukey’s multiple comparison test) (adjusted *P*-value <0.0001 and 0.0392, respectively). Individuals with *SDHx*-deficient tumors have significantly higher plasma succinate than *SDHx* carriers not harboring a tumor (adjusted *P*-value = 0.0408). *SDHx* carriers are individuals without current tumors (including prior resected tumor(s). (Panel B) AUROC curve analysis demonstrates that a cutoff of 3.69 μM is the optimal concentration to distinguish asymptomatic *SDHx* carriers from *non-SDHx* carriers. (Panel C) Asymptomatic *SDHx* carriers with *SDHA* (*n* = 3) (purple) and *SDHB* (*n* = 33) (dark blue) pathogenic variants have elevated succinate levels compared with *SDHC* (*n* = 2) (light blue) and *SDHD* (*n* = 2) (green) carriers (two-tailed Mann–Whitney test, *P*-value = 0.0255). All samples (including serial) from *SDHx* carriers were included in this analysis. (Panel D) Positive correlation of succinate concentrations with age for *non-SDHx* carrier females (Pearson *r* = 0.5437, two-tailed *P*-value = 0.0445, *n* = 14) and males (Pearson *r* = 0.2451, two-tailed *P*-value = 0.5963, *n* = 7) without tumors. Of note, the age range for the graphed cohort is 39–81 years old, and the full cohort ages ranged from 17 to 81. (Panel E) Scatterplot displaying a positive correlation of succinate concentrations with BMI in *non-SDHx* males (Pearson *r* = 0.6414, two-tailed *P*-value = 0.1205, *n* = 7) and females (Pearson *r* = 0.06939, two-tailed *P*-value = 0.8137, *n* = 14). Asterisks indicate statistical significance at the following levels: *P <0.05; **P <0.01; ***P <0.001; ****P <0.0001.

### *In vivo* plasma succinate positively correlates with tumor burden

As circulating succinate appears to be a specific biomarker for *SDHx* deficiency, we sought to assess whether quantitative levels were sensitive to tumor presence and burden. *SDHx* carriers with a tumor had a significantly increased (43.2%) mean succinate concentration compared with asymptomatic *SDHx* carriers without a tumor (6.69 and 4.76 μM, respectively, *P*-value = 0.0408), and two-way ANOVA confirmed a significant *SDHx* x tumor status interaction (*P* = 0.0157) (Fig. 2, Panel A; Supplementary Fig. 2). Furthermore, individuals with *SDHx*-deficient tumors (*n* = 29) had significantly elevated mean succinate concentrations (mean: 6.69 μM) in comparison to *non-SDHx* individuals with and without tumors (mean: 2.23 and 2.95 μM, respectively) (*P*-value = <0.0001 and <0.0001, respectively) (Fig. 2, Panel A). Tumors with *SDHC* hypermethylation had similar succinate levels to those driven by an *SDHx* germline pathogenic variant (data not shown). A cutoff of 2.76 μM of succinate had an ROC of 0.933 in distinguishing *SDHx-*deficient and proficient tumors (93.1% sensitivity, 86.4% specificity) (Fig. 3, Panel A). These findings are strongly correlated with the loss of SDHB immunohistochemical expression in 17 out of 19 assessable *SDHx*-deficient tumors (89.47%) (logistic regression OR of 0.6299, CI: 0.367–0.909). We found a significant effect of *SDHx* status on succinate levels (*P* value = 2.3 × 10^−9^), and this effect was larger in tumors than in non-tumors (*P*-value = 3.3 × 10^−3^) (Supplementary Fig. 2).

**Figure 3:**
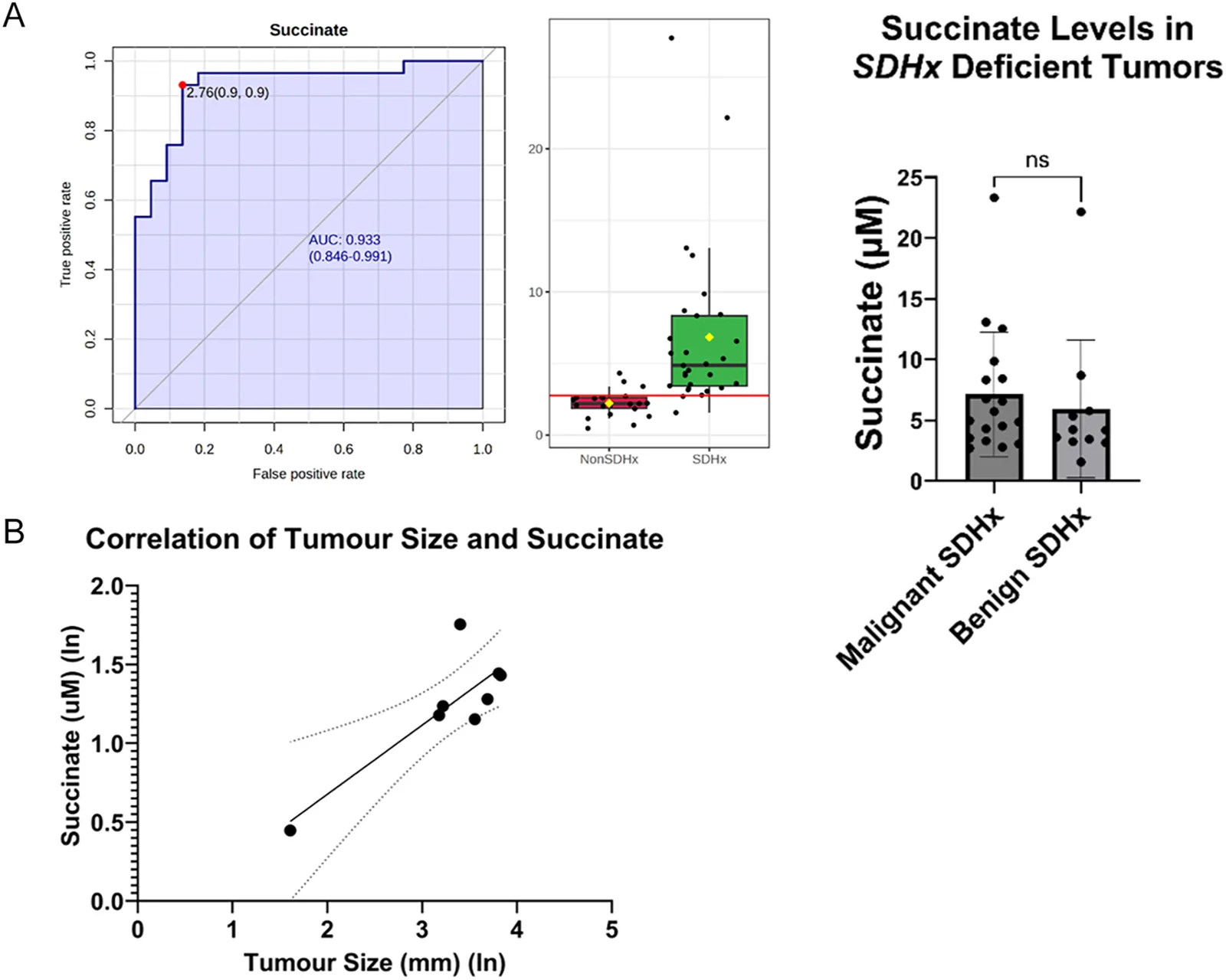
Elevated succinate in *SDHx*-deficient tumors and factors contributing to elevation. (Panel A) AUROC analysis between individuals with *SDHx*-deficient and *SDHx*-proficient tumors, illustrating the optimal cutoff of 2.76 μM of succinate with an ROC of 0.933. (Panel B) Positive correlation of plasma succinate concentrations (ln) with tumor size (ln) in *SDHx* deficiency (Pearson *r* = 0.8440, *R*^2^ = 0.7123, *P*-value = 0.0084; Spearman *ρ* = 0.619, *P*-value = 0.115) (*n* = 8). (Panel C) Succinate by tumor burden. Malignant *SDHx*-deficient tumors have similar succinate levels compared with *SDHx*-deficient non-malignant tumors (unpaired *t*-test with Welch’s correction, adjusted *P*-value = 0.5727) (malignant, *n* = 18; non-malignant, *n* = 11). Asterisks indicate statistical significance at the following levels: *P <0.05; **P <0.01; ***P <0.001; ****P <0.0001.

We next assessed the influence of tumor subtype, *SDHx* subunit, and tumor burden on succinate levels. Although limited by sample size, there was no difference in succinate levels between metastatic GIST and PPGL cases or non-metastatic cases of either tumor type (GIST, *n* = 12; PPGL, *n* = 17). Similarly, the small sample size limited assessment of tumor subtype (PCC, PGL, HNPGL, and GIST) on succinate levels in non-metastatic tumors. A marked difference in mean succinate level distribution per *SDHx* subunit was observed in individuals with *SDHx* non-metastatic HNPGLs, consistent with findings previously noted for *SDHx* carriers (*SDHA/B* vs *SDHC/D*, Mann–Whitney test, *P*-value = 0.0571). Succinate concentrations showed a positive ln–ln correlation with tumor size in individuals with non-metastatic or localized tumors (Fig. 3, Panel B) (Pearson, *R*^2^ = 0.7123, *P*-value = 0.0084), although the corresponding rank-based association did not reach significance (Spearman *ρ P*-value = 0.115). When individuals with *SDHx*-deficient tumors were grouped by tumor size (metastatic, non-metastatic >3 cm, and non-metastatic <3 cm), succinate levels progressively increased in relation to tumor burden and size. Metastatic tumors did not have significantly elevated succinate levels compared with non-metastatic tumors (*P*-value = 0.5727) (Fig. 3, Panel C).

### Plasma succinate is a candidate biomarker for disease progression

Three individuals with *SDHx*-deficient tumors, including WT GIST, had serial plasma succinate levels measured over months (1–13 months) (Fig. 4A-D, Supplementary Table 1). Rising succinate levels correlated with disease progression on imaging and biochemical testing in all cases, heralding disease progression. One case is illustrated in Fig. 4, where despite stable levels of plasma normetanephrine over time (Fig. 4B), metastatic spread in bone in an individual with an *SDHA*-associated metastatic paraganglioma trended with succinate levels and preceded detection on molecular imaging and subsequent elevation of plasma normetanephrine. Rising succinate levels were similarly linked with progression of one individual with metastatic GIST (ID37), for which we have limited clinical biomarkers of progression, and a case of metastatic PCC (ID116) (Fig. 4C and D).

**Figure 4:**
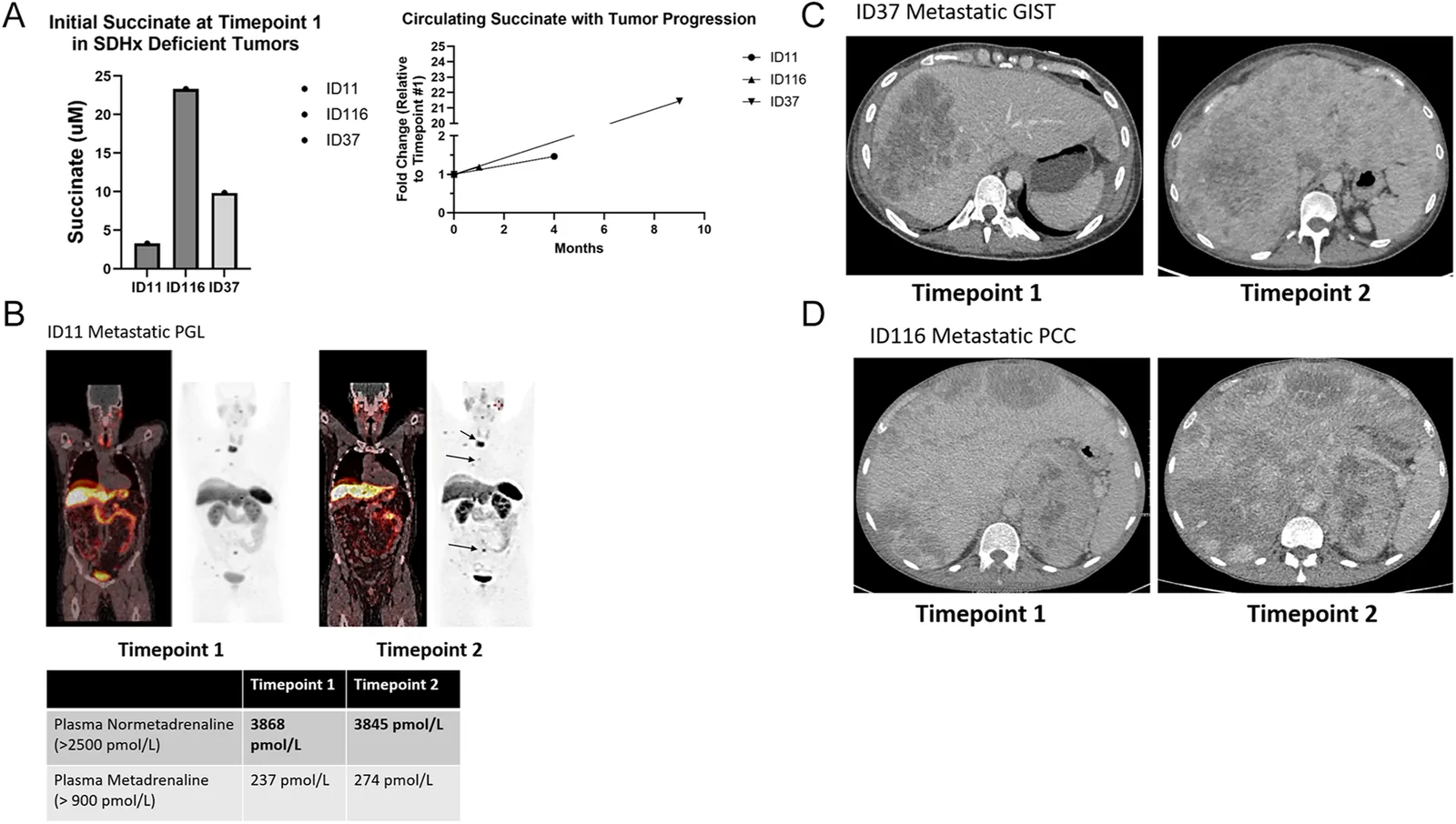
Progression of tumor burden tracks with succinate elevation. All three individuals with *SDHx*-deficient tumors demonstrated temporal increases in circulating succinate corresponding to disease progression. (A) Spaghetti plot of all participants and (B, C, D) individual plots of succinate levels. ID37 WT GIST; ID116 PCC; ID11 para-aortic PGL. (B) For ID37, at timepoint 1, metastases were identified, and the patient underwent therapy with regorafenib and temozolomide. Despite treatment, in a 10-month time period, disease progression of metastases (shown on MRI imaging) occurred with a concurrent increase in succinate (21.4-fold). (C) ID116 presented to the clinic with fulminant disease and was found to have metastatic spread to the lung, liver, and rib (MRI imaging). Despite modestly elevated plasma metanephrine levels at 1,043.7 pmol/L, succinate levels increased over a 7-day period. The patient ultimately succumbed to the disease one month later due to progressive enlargement of metastases. (D) At the time of original blood collection, ID11 had a para-aortic paraganglioma, which had metastasized to bone and was treated with four cycles of Lutathera (^177^Lu-DOTATATE). The first plasma succinate level was checked 6 months after completing 4 cycles of Lutathera treatment (timepoint 1). A 1.5-fold increase in succinate was detected at an interim 5-month timepoint (timepoint 2), reflecting disease progression, which was later discovered on MRI, ^68^Ga-DOTATATE PET/CT, and fluorodeoxyglucose (FDG)-PET/CT imaging. Notably, plasma normetanephrine levels remained stable from timepoint 1 to several months after succinate elevation was noted at timepoint 2, indicating that succinate may be an early indicator of tumor progression.

### *Sdhb*-deficient mouse model recapitulates tissue-specific metabolic rewiring despite stable plasma succinate concentration

With an emphasis on *SDHx* deficiency driving succinate accumulation, central carbon metabolism was assessed on two tyrosine hydroxylase promoter-driven *Sdhb-*deficient murine models: conditional *Sdhb* knockout model (*Th-Cre; Sdhb^Flox/Flox^*) and a two-hit model with a pathogenic germline variant and somatic loss of heterozygosity (*Th-Cre; Sdhb^R46Q/Flox^*). There was no significant difference in succinate in metabolically active organs (brain, gastrocnemius, and heart samples) between different genotype groups. Succinate and acetyl-CoA concentrations were significantly elevated in *Th-Cre; Sdhb^R46Q/Flox^* mice compared with WT mice (*P*-value = 0.0191 and 0.0034, respectively), in line with variable SDHB protein expression loss (Supplementary Fig. 3), and other TCA metabolites were not significantly altered (Fig. 5A). Furthermore, acetyl-CoA was significantly elevated in the *Th-Cre; Sdhb^Flox/Flox^* model in comparison to WT adrenals (*P*-value = 0.0321). Glycolytic metabolites were significantly reduced in *Th-Cre; Sdhb^R46Q/Flox^* mice, suggesting the utilization of alternative metabolites to complement the TCA cycle (Fig. 5B).

**Figure 5:**
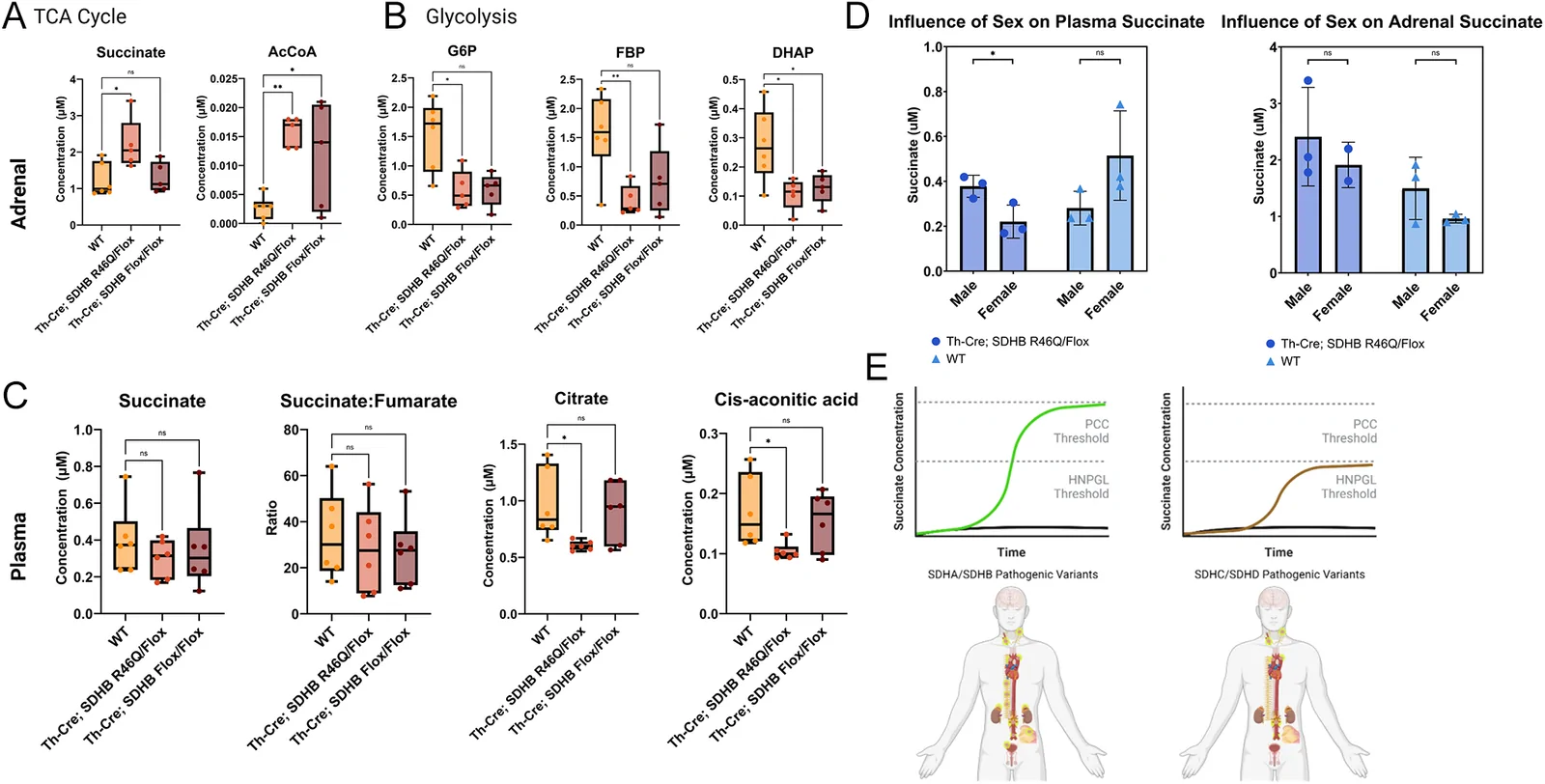
Plasma and adrenal tissue metabolomics in *Sdhb*-deficient murine model. Central carbon metabolism analysis of adrenal (Panels A and B) and plasma (Panel C) samples of WT (*n* = 6), *Th-Cre; Sdhb^R46Q/Flox^* (*n* = 6), and *Th-Cre; Sdhb^Flox/Flox^* (*n* = 6). (Panels A and B) The adrenals of *Th-Cre; Sdhb^R46Q/Flox^* mice demonstrate significantly reduced glycolytic metabolites (e.g. G6P, glucose 6-phosphate; F6P, fructose 6-phosphate; DHAP, dihydroxy-acetone-phosphate) with elevated acetyl CoA and succinate (one-way ANOVA). Only one glycolytic intermediate, DHAP, was reduced, and acetyl CoA (AcCoA) was elevated in the *Th-Cre; Sdhb^Flox/Flox^* mice. (Panel C) Succinate and succinate-to-fumarate ratios were not significantly different between WT and the two Sdhb-deficient mouse models; however, citrate and cis-aconitic acid were in the *Th-Cre; Sdhb^R46Q/Flox^* model. (Panel D) Subgroup analysis by sex identified that male *Th-Cre; Sdhb^R46Q/Flox^* mice had significantly elevated circulating succinate compared with female mice. (Panel E) Conceptual framework of succinate thresholds per SDHx subunit. Higher relative succinate concentrations in SDHA/B deficiency reach the physiologic set point in each tissue, leading to anatomically distributed disease, whereas SDHC/D deficiency results in isolated disease.

Neither *Sdhb* two-hit model had elevated plasma succinate nor succinate-to-fumarate ratios, although citrate and cis-aconitate were reduced in *Th-Cre; Sdhb^R46Q/Flox^* mice compared with WT mice (*P*-value = 0.0354 and 0.0412, respectively). There were no significant metabolites in the pentose phosphate pathway or glycolysis (Fig. 5C). Plasma succinate in females was markedly higher than in males in WT mice, as in the patient cohort; however, the reverse was true in adrenal tissue, which may reflect physiological differences in succinate regulation at the tissue level and the influence of sex hormones on metabolite balance. There was a similar pattern of succinate concentrations between the sexes in the adrenals of *Th-Cre; Sdhb^R46Q/Flox^* mice; however, males harbored significantly elevated plasma succinate levels (*P*-value = 0.0370) compared with females (Fig. 5D), suggesting the *Sdhb* genetic state and hormones may influence this balance.

## Discussion

This prospective study consisted of 122 well-phenotyped patients (half of whom were *SDHx* carriers) with either a PPGL and/or germline predisposition to a PPGL, with genetic testing and regular long-term genotype-guided surveillance imaging throughout the course of the study. Consistent with recent investigations (15), this study found that succinate was elevated in both *SDHx* carriers and individuals bearing an *SDHx*-deficient tumor, illustrating that succinate is a sensitive plasma biomarker for identifying *SDHx* carrier status and enzyme deficiency. Lamy *et al.* identified that serum succinate in tumor-free and symptomatic *SDHx* carriers were 7 and 14 μM, respectively (11). Our study findings identified that *SDHA/B* carriers with and without tumors have higher circulating succinate levels than *SDHC/D* carriers with and without tumors. Analysis of data from a previously published tumor metabolomic study from Richter *et al.* (16) suggests that there may be differences in succinate concentrations depending on the *SDHx* subunit. Furthermore, Gebhardt *et al.* highlighted this trend in *SDHx* carriers with tumors pre- and post-surgery, but analysis was not performed solely on unaffected carriers (10).

Elevated succinate levels in asymptomatic carriers leads to a hypothesis that i) there may be *SDHx* null cells (from which succinate leaks into the plasma) within tissues not associated with SDHx-associated tumors and ii) there may be tissue specific thresholds for succinate-induced tumorigenesis, whereby elevation above a threshold initiates cell proliferation in certain cell types but in other tissues is not pro-oncogenic (Fig. 5E). Interestingly, similar theories have been suggested for HIF-2α-related oncogenesis in renal cell carcinoma and PPGL (14, 17), and there is a selective pressure for a specific degree of beta-catenin signaling dysregulation with somatic mutations in the APC tumor suppressor gene (18). Succinate plays a critical role in oxygen sensing in the carotid body and has been found in high concentrations compared with adrenal medulla, where succinate mediates the release of catecholamines (19). Within this schema, small elevations in succinate due to *SDHC* and *SDHD* pathogenic variants would predispose to HNPGL tumor development but would not reach the physiologic set point of adrenal medulla. In contrast, *SDHA* and *SDHB* deficiency may already reach the maximum physiological succinate threshold in both carotid body and adrenal medulla. This study provides evidence of a succinate threshold model, proposed by Bayley *et al.* (20), where systemic biomarker detection depends on a balance between tumor production and tissue clearance. The mouse data suggest that a ‘two-hit’ event at the tissue level is sufficient for local metabolic disruption but may not always exceed the physiological clearance threshold required for serum detection. Critically, there were no significant differences in circulating plasma concentrations in asymptomatic carriers with missense or truncating *SDHB* pathogenic variants, and this study was underpowered to assess plasma succinate levels between individuals carrying tumors with *SDHx* missense and loss-of-function (LOF) variants.

In addition to *SDHx* genotype, we found that age (older) and sex (female) in the control cohort trended with circulating succinate levels, which warrants further investigation, especially in the pediatric population. A recent study of 93 individuals (42 of which were *SDHx* carriers) found that male sex was an independent risk factor for serum succinate levels; however, this analysis was performed across the entire study population, including those with and without PPGLs (21). There is a need to further define the normal distribution of succinate levels in the general population and to determine if succinate is detectable in plasma from birth or childhood or whether it develops and accumulates over time. We cannot exclude the contribution of succinate-producing gut microbiota (primarily *Bacteroides* and *Prevotella*) (22, 23), and this warrants consideration in future large cohorts.

A novel discovery in this study was that individuals bearing an *SDHx-*deficient tumor, both germline- and somatically driven PPGLs and GISTs, had significantly elevated succinate in comparison to asymptomatic *SDHx* carriers. Circulating levels positively correlated with tumor burden, in line with recent studies (10). Uniquely, succinate levels tracked with disease progression in three individuals with multiple samples. These results have implications for individuals with *SDHx-*deficient tumors, which warrant close surveillance and are especially important for individuals with unresectable tumors and unknown somatic genetic status.

Plasma metabolomics provides a rapid and inexpensive non-invasive tool for stratifying the likelihood of a genetic diagnosis of *SDHx* deficiency and could be applied in resource-poor settings where access to DNA sequencing may be limited. In addition, if genetic testing for a suspected *SDHx* PGV is unexpectedly negative, the presence of elevated plasma succinate would be an indicator for further genetic analysis such as *SDHC* promoter hypermethylation, as we have shown, and alterations in *SLC25A10*, *SUCNR1*, and *SDHAF1-4*. Likewise, in 3 cases of progressive disease, succinate tracked with disease progression and may be utilized as a surrogate marker of progression or response to therapeutics with further validation from additional studies. The finding of elevated succinate in *SDHx* carriers suggests that succinate accumulation may be either a result of accumulating cells with biallelic *SDH*x loss or potentially an effect of haploinsufficiency independent of biallelic inactivation. Although long-term clinical studies are needed to assess whether succinate levels in *SDHx* carriers reflect currently clinically undetectable *SDHx*-deficient cells, an *Sdhb* murine model provides an opportunity to assess the role of biallelic inactivation on succinate accumulation.

Central carbon metabolism analysis in plasma and adrenals of two *Sdhb*-deficient murine models revealed that the adrenals of both models had marked metabolic rewiring resulting in reduced glycolytic intermediates and significant succinate accumulation in the *Th-Cre; Sdhb^R46Q/Flox^* mice (representative of patients with germline inheritance). Succinate levels in the plasma were unexpectedly similar between the Sdhb models and WT mice; however, *Th-Cre; Sdhb^R46Q/Flox^* males had significantly elevated circulating succinate levels compared with female counterparts. Clinical cohorts have demonstrated that male sex is associated with more aggressive disease, including more frequent metastatic presentation at diagnosis and shorter survival (24, 25). These results may be explained by i) insufficient quantitative biallelic inactivation of *Sdhb* in TH + cells of the adrenal medulla that leads to succinate accumulation in plasma and/or ii) differences between mice and humans in terms of regulation of cellular efflux of succinate or clearance of circulating succinate levels. Differences in organ size and metabolic rate between mice and humans may be the key to these unexpected results.

Succinate plays a critical role in anoxia sensing within tissues such as the carotid body and adrenal medulla and, in response to stimuli such as ischemia, lactic acidosis, or hyperglycemia, can be transported extracellularly in its protonated monocarboxylic form via MCT1 (26). Paracrine signaling has been identified to have a significant role in muscular remodeling (26) and in promoting brown fat thermogenesis (27). Hence, these target organs may serve as a sink for circulating succinate. While these mechanisms may be present in the *Sdhb* WT state, we propose that these regulatory processes may be overburdened in *Sdhb* monoallelic inactivation, leading to succinate accumulation. There are species-specific differences in SUCNR1 that may explain the difference in human and mouse data: SUCNR1 is expressed at higher basal levels and in a broader number of tissues in mice versus humans (Bgee dataset, Supplementary Fig. 4) (28, 29), which may lead to different phenotypes in the two species. As such, high metabolic rate organs may serve as a metabolic ‘sink’, clearing circulating succinate before it can accumulate to levels observed in our patient cohort.

In summary, this prospective cohort study identified plasma succinate as a promising specific and sensitive biomarker of *SDHx* variant status, which tracks disease progression for individuals bearing tumors. Studies in an *Sdhb*-deficient mouse model support and validate the direct link between *SDHx* deficiency and circulating succinate levels and shed light on species differences in succinate regulation that may reflect the challenge in *SDHx*-deficient PPGL model development.

## Supplementary materials



## Declaration of interest

The authors declare that there is no conflict of interest that could be perceived as prejudicing the impartiality of the work reported.

## Funding

The authors acknowledge the important contributions made by collaborators, funders, and those maintaining research infrastructure. YC receives funding from the NIH Oxford-Cambridge Scholars Program and the Gates Cambridge Scholars Program. RC is supported by the Cambridge NIHR Biomedical Research Centre (NIHR203312). *The views expressed are those of the authors and not necessarily those of the NIHR or the Department of Health and Social Care.*

## Author contribution statement

RTC, ERM, EG, and YCC conceived and designed the study. YCC, RTC, JM, and FD collected and prepared human plasma samples. YCC, IA, JFG, EG, RTC, and ERM analyzed human sample data. YCC, XX, TA, and HW were responsible for the collection and/or analysis of mouse sample data. All authors were involved in the final preparation, review, and approval of the manuscript.

## Data availability

Clinical information was extracted from electronic health records at the Cambridge University Hospitals NHS Foundation Trust. Data generated and analyzed in this study are included in this article or supporting documentation. Additional information is available from the corresponding author upon reasonable request.

## Participant recruitment and clinical data

Patients were recruited and consented to the Molecular Pathology of Human Genetic Disease Study (South Birmingham REC CA/125) at Addenbrooke’s Hospital. Eligible participants provided informed consent at Addenbrooke’s neuroendocrine tumor clinic during the prospective study (2020–2024), in accordance with the principles of the Declaration of Helsinki and clinical data was collected from patient records. This study was conducted according to the National Institute for Healthcare Research Good Clinical Practice guidelines. 

## AI disclosure

No generative artificial intelligence (AI) tools were used in the preparation of the written or visual content of this manuscript.
