# Supplementary material for: Investigating the clinical utility of plasma succinate with insights from an Sdhb-deficient murine model
Source: Endocr Relat Cancer. 2026 Oct 13;33(10):e260323. doi: 10.1530/ERC-26-0323 (PMC13695350; doi:10.1530/ERC-26-0323)
Supplement: Supplementary file 1 [file ERC-26-0323_supplementary_materials.pdf]

SUPPLEMENTARY MATERIALS

Pages 2-6…Supplementary Methods

Pages 8…Supplementary Results

Pages 9…Supplementary Figures

Pages 12…Supplementary Tables

Supplementary Methods

Definition of benign and metastatic PPGL

A PPGL diagnosis was defined as a positive biochemical screening test and detection of a lesion/tumor on clinical imaging and/or histopathology. Metastatic disease was defined by either 1) lesions within the bone, liver, lung, peritoneum, or lymph nodes by clinical imaging (MRI, CT, or radionuclide imaging), following WHO guidelines, or histopathological diagnosis in adjacent resected lymph nodes or 2) surgical site recurrence, identified by biochemistry and clinical imaging [10].

Mouse husbandry and genotyping

Animals were housed at the NIH and treated in accordance with the Institutional Animal Care and Use Committee at the Eunice Kennedy Shriver National Institute of Child Health and Development (NICHD). Genotyping of tail snips were performed to evaluate the *Sdhb*^{Flox} allele (F primer TCA CCT TCC TGT ACC TTT GTA GTG, R primer GGT CAC ATA GCC AGT GAC TT), *Sdhb*^{R46Q} allele (F primer ATA AGT TCT GTG GAG CGA AGG TTC, R primer CGA CAG GCC TGA AAC TTG AAA AAG), and Cre transgenic allele (F primer GGA AAA TGC TTC TGT CCG TTT G, R primer TTG GTC CAG CCA CCA GCT TG). Tail snip DNA was extracted using Qiagen's DNeasy Blood and Tissue Kit (Cat No 69506) using the manufacturer's protocol. For DNA amplification, 1-2 µL of DNA was used in combination with OneTaq Quick-Load 2x Master Mix with Standard Buffer (New England BioLabs, Cat no M0486L), H_2O, and gene primers. PCR products were visualized on 1% w/v agarose (SYBR

Safe DNA Gel Stain, Invitrogen lot no 2203142) and imaged on the Image Lab Touch Software on a BioRad ChemiDoc MP Imaging System.

Generation of Sdhb murine model

Sdhb^{R46Q} murine model was developed through CRISPR/cas9-mediated homologous recombination utilizing a donor plasmid of the *Sdhb* R46Q point mutation (CGA to CAA) into B6:129-mixed background mouse embryonic stem cells (ESC). The positive ESC colony was selected through neomycin (the plasmid contained an insertion of a reverse-oriented neomycin resistance gene cassette flanked by loxP sites) and injected into mouse blastocysts. After breeding founder mice with EIIα-Cre transgenic mice to remove loxP-neo-loxP, *Sdhb*^{R46Q} were confirmed with PCR and Sanger sequencing. In addition, B6:129-*Sdhb*^{tm1Nju} mice were imported from the Model Animal Research Centre of Nanjing University. The FRT-neo-FRT cassette harbored by these mice was genetically removed with Flpe transgenic mice, resulting in *Sdhb*^{Flox} mice carrying loxP sites flanking exons 3-6. Th-Cre; *Sdhb*$^{Flox/+}$ mice were generated by crossing B6 mice expressing Cre under the control of tyrosine hydroxylase (TH) (Jackson Laboratory, B6.Cg-7630403G23Rik$^{Tg(Th\text{-}cre)1Tmd}$/J, Strain #008601) with *Sdhb*$^{Flox/+}$. A two hit model of Sdhb were generated by crossing *Sdhb*^{R46Q} mice with Th-Cre; *Sdhb*$^{Flox/+}$ mice.

Collection of mouse plasma and tissue

Murine blood (100 μL) was collected in EDTA tubes from the facial vein of 6 adult WT C57BL/6 and 12 transgenic *Sdhb* deficient mice and immediately placed on ice. Blood was processed using the same method as human samples, as described in the main manuscript. Mouse

tissues (bilateral adrenal glands, brain, gastrocnemius, and heart) were isolated from 6 adult WT C57BL/6 and 12 transgenic *Sdhb* deficient mice (6 Th-Cre; *Sdhb*$^{R46Q/Flox}$, 6 Th-Cre; *Sdhb*$^{Flox/Flox}$) using the Stemi 508 stereo microscope with boom stand and Axiocam ERc 5s standalone color camera (Germany) after CO_2 asphyxiation. Tissue weights were measured prior to snap freezing in liquid nitrogen for metabolic concentration normalization and samples were stored at -80°C prior to Liquid Chromatography/Mass Spectrometry (LC/MS).

Liquid chromatography mass-spectrometry and targeted metabolomics of human plasma

Plasma was thawed on ice, diluted 1:10 cold metabolites extraction solvent (Methanol: Acetonitrile; 75:25) (−20°C), vortexed for 10 minutes at 4°C and then centrifuged at 16,000xg for 10 min at 4°C. Supernatant was transferred to LC/MS vials and stored at −80°C. Samples were run on the ZIC-pHILIC column (SeQuant; 150 mm × 2.1 mm, 5 µm; Merck) and Thermo Vanquish Flex ultra-high-performance liquid chromatography (UPLC) system coupled to Orbitrap Exploris 240 Mass Spectrometer (Thermo Scientific) at a resolution of 120,000 at 200 mass/charge ratio (m/z), electrospray ionisation, and polarity switching mode to enable both positive and negative ions across a mass range of 67–1000 m/z. 5 µL of plasma extracts were injected, and the compounds were separated with a mobile phase gradient of 15 min, starting at 20% aqueous (20 mM ammonium carbonate adjusted to pH 9.2 with 0.1% of 25% ammonium hydroxide) and 80% organic (acetonitrile) and terminated with 20% acetonitrile. Flow rate and column temperature were maintained at 0.2 ml/min and 45 °C, respectively, for a total run time of 27 min. All metabolites were detected using mass accuracy below 1 ppm. Thermo Xcalibur 4.4 was used for data acquisition. The peak areas of metabolites were determined using Thermo TraceFinder 4.1 software, where metabolites were identified by the exact mass of the singly

charged ion and by known retention time on the using an in-house MS library built by running commercial standards of all detected metabolites.

Reversed-Phase Ion-Pairing LC-MS/MS Assay for Tissue Central Carbon Metabolites of Mouse Plasma and Tissues

The TCA cycle, pentose phosphate pathway, and oxidative phosphorylation metabolite concentrations from plasma, adrenal, and metabolically active organs (brain, skeletal muscle (e.g., gastrocnemius), heart)[16] of 18 mice with various genotypes (WT, *Th-Cre; Sdhb*$^{R46Q/Flox}$, *Th-Cre; Sdhb*$^{Flox/Flox}$) were isolated with methods described above. Tissue samples were added to 400 µL chilled 80% methanol in H_2O and homogenized with ceramic beads. After centrifugation at 3,000xg for 30 min at 4°C, supernatant was added to stable isotope labeled internal standards methanol solution, dried and then reconstituted in 3% (v/v) methanol in H_2O. Each sample was injected for reversed-phase ion-pairing LC-MS/MS analysis: liquid chromatography was carried out on a Synergi Hydro-RP C18 column. Quantitation of tissue CCM was carried out using Xcalibur™ Quan Browser (Thermo Scientific). All reference target compounds of central carbon metabolites (CCM) were purchased from Sigma-Aldrich (St. Louise, MO).

The stable isotope labeled internal standards (SI-CCM) were $^{13}C_3$-lactate, $^{13}C_4$-succinic acid, obtained from Cambridge Isotope Laboratory (Andover, MA) as well as $^{13}C_6$-glucose-6-phosphate and $^{13}C_6$-fructose-1,6-diphosphate purchased from Medical Isotopes, Inc. (Pelham, NH). All CCM and SI-CCM analytical standards have reported chemical and isotopic purity ≥ 98%. They were used without further purification. OmniSolv® LC-MS grade acetonitrile and methanol were obtained from EMD Millipore (Billerica, MA). Tributylamine (TBA), LC-MS

grade acetic acid and formic acid were purchased from Fisher Scientific (Hampton, NH). All chemicals and solvents used in this study were HPLC or reagent grade unless otherwise noted.

The LC-MS/MS method employed was adapted from previous publications [28,29]. Tissue samples were added to 400 µL chilled 80% methanol in H_2O and homogenized with ceramic beads on the Bead Ruptor™ (Omni International, Kennesaw, GA). After centrifugation at 3,000xg for 30 min at 4°C, 50 µL supernatant was added to 80 µL of 10 µM SI-CCM methanol solution, dried with the SpeedVac® vacuum concentrator (ThermoFisher Scientific, Waltham, MA) and then reconstituted in 60 µL 3% (v/v) methanol in H_2O. Ten µL of each sample was injected for reversed-phase ion-pairing LC-MS/MS analysis. Analysis was performed using a Thermo TSQ™ Quantiva triple quadrupole mass spectrometer (Thermo Scientific, San Jose, CA) coupled with a NexeraXR LC system (Shimadzu Scientific Instruments, Columbia, MD). Both the HPLC and mass spectrometer were controlled by Xcalibur™ software (Thermo Scientific). Reversed-phase ion-pairing liquid chromatography was carried out on a 100-mm long x 2.1-mm i.d. Synergi Hydro-RP C18 column with 2.5 µm particles and 100 Å pore size (Phenomenex, Torrance, CA) and kept in 40 °C. The mobile phase, operating at a flow rate of 200 µL/min, consisted of 10 mM TBAA in water as solvent A and methanol as solvent B. For the analysis of CCM and SI-CCM, a linear gradient stayed at B/A solvent ratio 3:97 for 3 min, then changed the B/A solvent ratio from 3:97 to 80:20 in 14 min. After washing with 98% B for 3 min, the column was re-equilibrated with a mobile phase composition B/A of 3:97 for 10 min prior to the next injection. Quantitation of tissue CCM was carried out using Xcalibur™ Quan Browser (Thermo Scientific). Calibration curves for each CCM were constructed by plotting CCM/SI-CCM peak area ratios obtained from calibration standards versus CCM concentrations and fitting these data

using linear regression with 1/X weighting. The CCM concentrations in samples were then interpolated using this linear function.

Supplementary Results

Correlation of succinate concentration with sex, age, and BMI

Elevated levels of circulating succinate have been found in the cardiovascular disease [12], type 2 diabetes [13], and obesity [14]. Further, Bowman and colleagues reported that reduced complex II activity with reduced expression of succinate dehydrogenase complex is associated with ageing fibroblasts [15]. To examine the impact of these and other factors on succinate levels, we assessed the correlation of succinate with age, BMI, and sex in participants with negative germline genetic testing. A gender-stratified analysis revealed a significant positive correlation between age and succinate among females (Pearson R = 0.5437, p-value = 0.0445). In contrast, no such relationship was observed for males (Pearson R = 0.2451, p-value = 0.5963). BMI had a trending but non-significant positive association (Pearson R = 0.3214, p-value = 0.1205) (**Figure 2, Panel D, E**).

Supplementary Figures

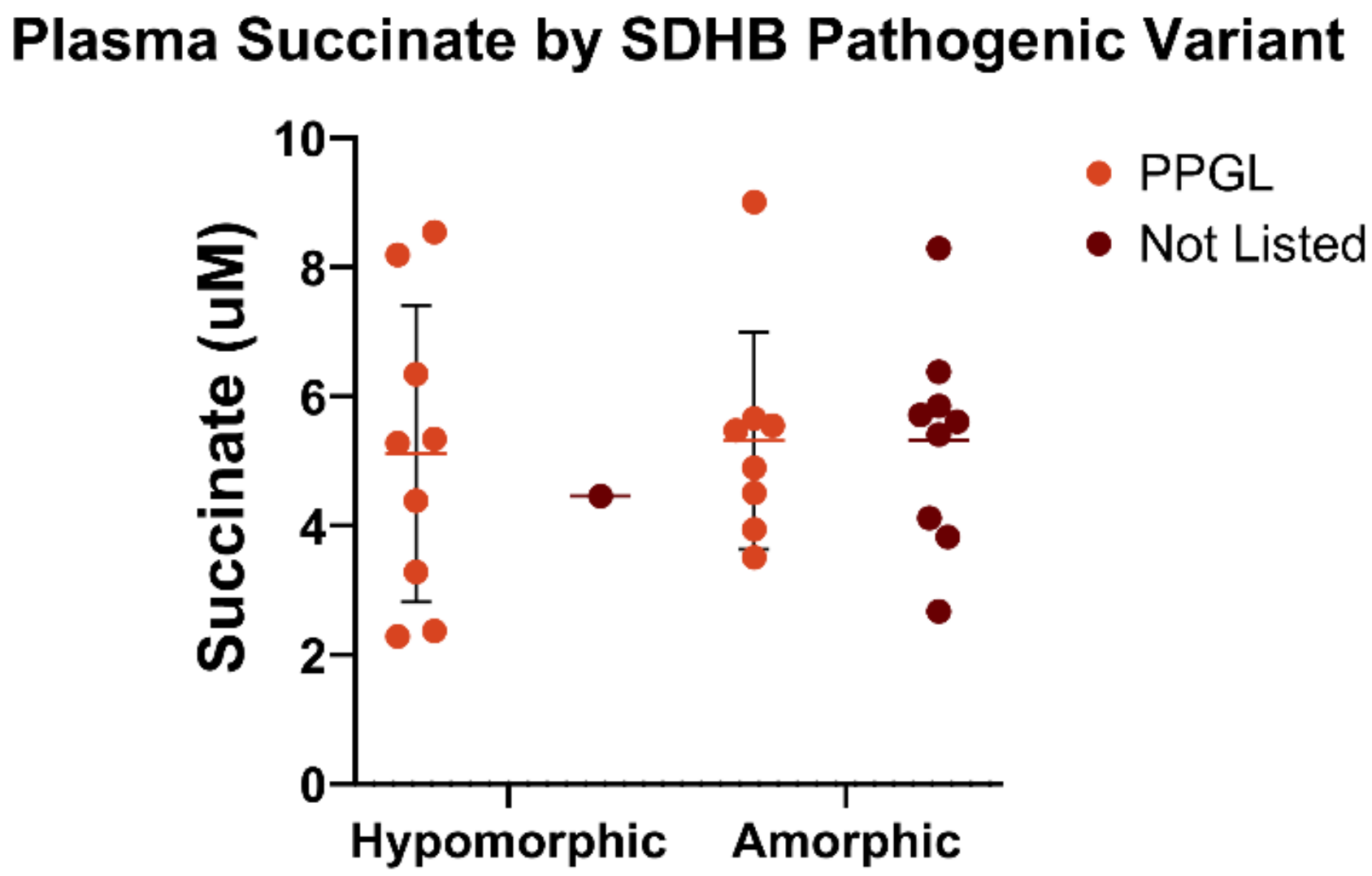

Supplementary Figure 1. Circulating succinate levels in *SDHB* carriers by pathogenic variant. *SDHB* carriers were categorized by pathogenicity (hypomorphic and amorphic) and the PPGL subtype predominance, as outlined by [11]. The cohort did not include variants that predisposed to HNPGLs. Not listed includes one missense variant *SDHB* p.Pro197Arg, whole exon deletions and frameshift variants. Succinate levels did not vary between PPGL predominant hypomorphic and amorphic variants (2way ANOVA, p-value = 0.9703) nor variants with an unknown PPGL subtype predominance (2way ANOVA, p-value = 0.8920)

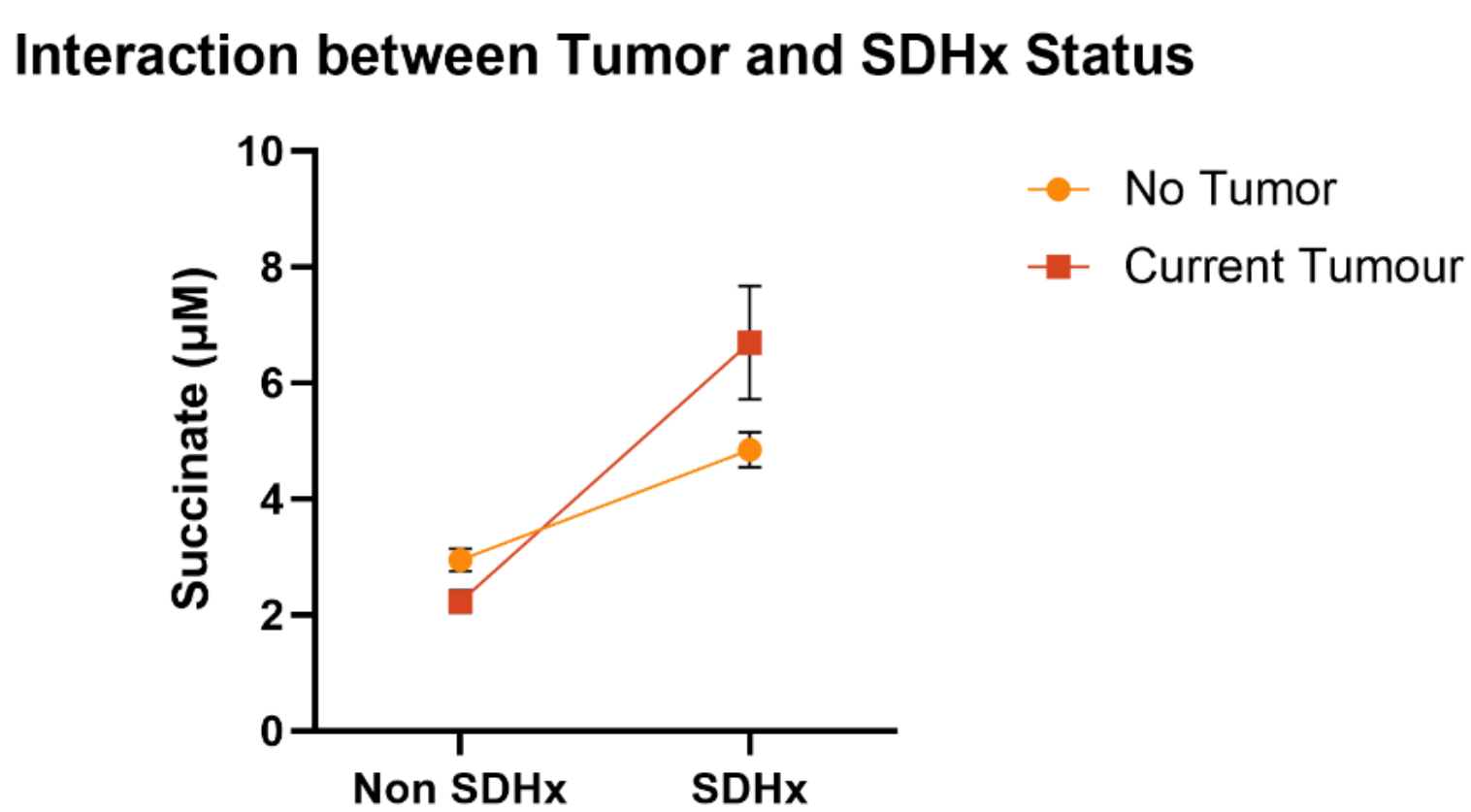

Supplementary Figure 2. Succinate levels are influenced by interaction of *SDHx* and Tumor status. Plasma succinate concentrations were compared across *SDHx* status (*non SDHx* vs *SDHx*) and tumor status (no current tumor vs current tumor) by two-way ANOVA using Type 1 (sequential) sums of squares (n = 122; *non SDHx*/no tumor, n = 39; *non SDHx*/current tumor, n = 22; *SDHx*/no tumor, n = 32; *SDHx*/current tumor, n = 29). Analysis revealed a significant main effect of *SDHx* status (F = 32.93, p-value = 7.5×10^{-8}) and a significant *SDHx* x tumor status interaction (F = 6.01, p-value = 0.0157). The magnitude of the increase in succinate associated with *SDHx* status was significantly more pronounced in the current tumor group (orange) compared to the no tumor group (yellow), indicating the effect of an *SDH*x pathogenic variant is amplified by the presence of a tumor. Points represent group means; error bars represent SEM.

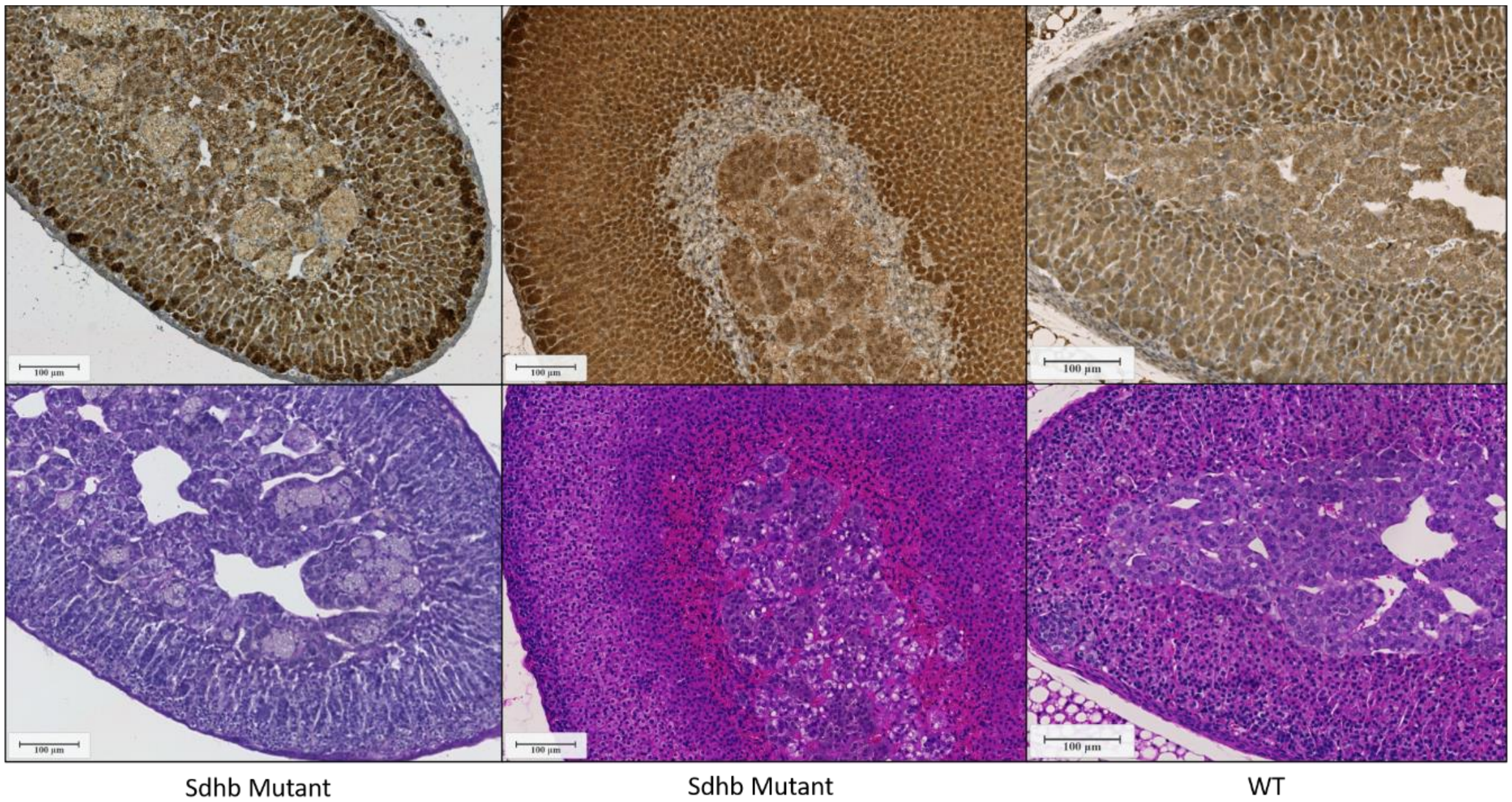

Supplementary Figure 3. SDHB protein loss in *Sdhb* deficient murine model. Representative images shown. Histologic sectioning, staining with hematoxylin and eosin and SDHB demonstrates patchy reduced SDHB expression within *Sdhb* mutant adrenal glands. Scale bar at 5x (100 µm) provided in images. Adrenal glands were isolated using the ZEISS Stemi 508 stereo microscopy, fixed in 4% PFA and embedded in in paraffin. Slides underwent H&E staining through standard protocols with Mayer's modified hematoxylin and Eosin Y solution with phloxine. For immunohistochemistry, slides were placed in citrate pH 6.0 Antigen Retriever Buffers (MilliporeSigma, cat no SIGC9999), 1:300 SDHB primary antibody (Sigma Aldrich, HPA002868) incubation in Renoir Red (Biocare Medical, cat no PD904) overnight, and labelling with Mach 4 polymer (MACH 4 Universal HRP-Polymer, BIOCARE MEDICAL, cat no M4U534G).

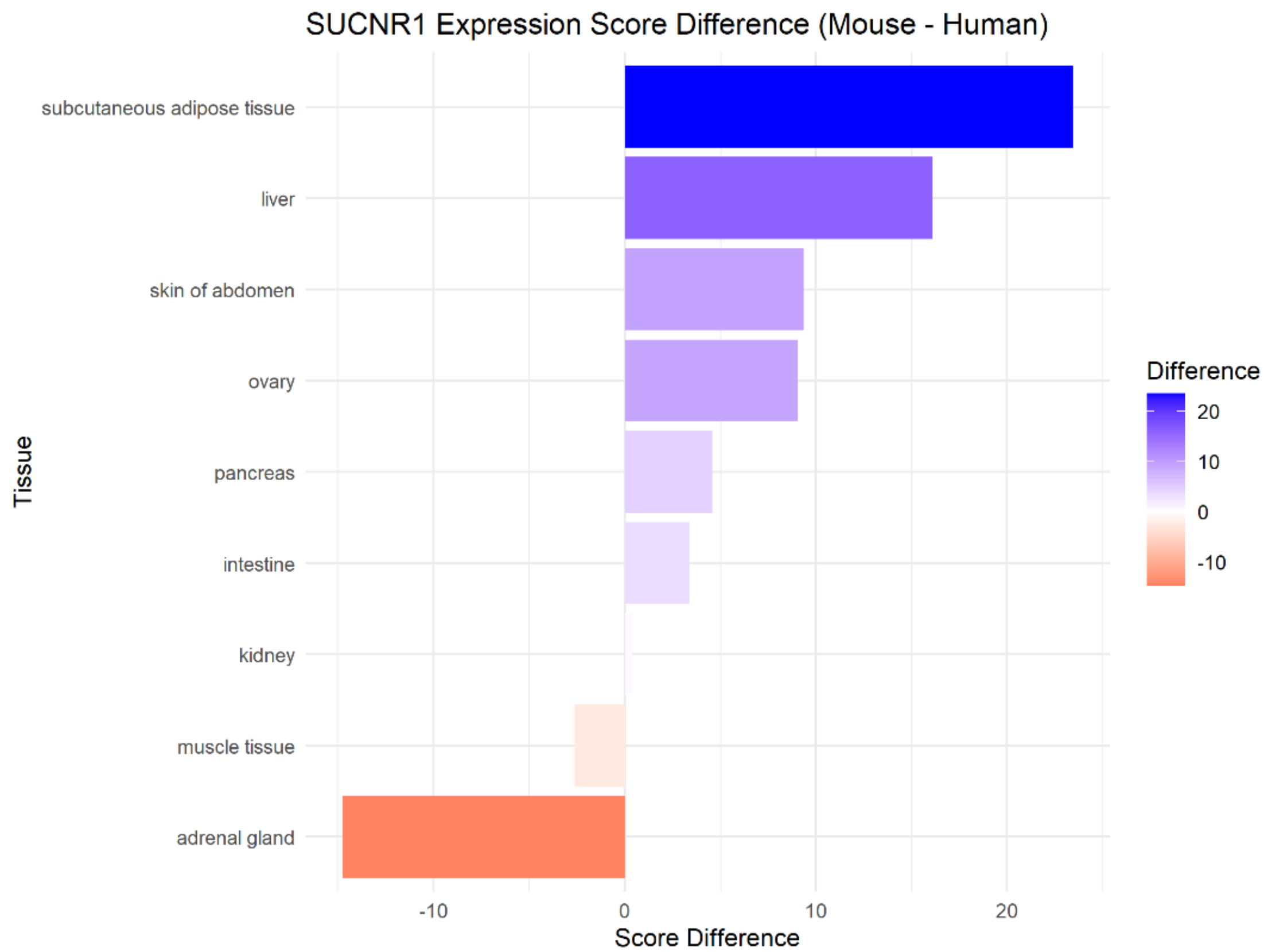

Supplementary Figure 4. Difference in SUCNR1 expression score across shared tissues of homo sapiens and mus musculus using the Bgee dataset. Bar chart depicting the difference in *Sucnr1*/*SUCNR1* expression score (Mouse-Human) across tissues common to both species for direct cross-species comparison, generated from the Bgee gene expression database (Release 15.2) (human, Ensembl ID ENSG00000198829; mouse, Ensembl ENSMUSG00000027762)[26, 27]. For each species, expression calls with a "present" status and gold/silver level confidence were retained. Bars represent score difference for each tissue, ordered from most negative to most positive. Tissues with a positive difference (blue) show higher expression in mus musculus, while tissues with a negative difference (red) show higher expression in homo sapiens. Of 28 shared tissues, n=9 were selected for comparison on this graph.

Supplementary Tables

Table 1. Circulating succinate across timepoints for patients with *SDHx* deficient tumors

Participant ID	Succinate at timepoint 1	Succinate at timepoint 2	Fold Change (relative to timepoint 1)	Months between timepoint 1 and 2 (months)
ID11	3.30	4.84	1.46	4
ID37	9.87	211.56	21.44	9
ID116	23.32	27.72	1.19	0 (7 days)
